# The influence of a doppler ultrasound in arteriovenous fistula for dialysis failure related to some risk factors

**DOI:** 10.1590/2175-8239-JBN-2019-0080

**Published:** 2020-04-27

**Authors:** Jocefábia Reika Alves Lopes, Ana Lígia de Barros Marques, João Antonio Correa

**Affiliations:** 1 Faculdade de Medicina do ABC, Santo André, SP, Brasil.; 2 Universidade Federal do Maranhão, Imperatriz, MA, Brasil.

**Keywords:** Renal Insufficiency, Chronic, Renal Dialysis, Arteriovenous fistula, Ultrasonography, Doppler, Insuficiência Renal Crônica, Diálise Renal, Fístula Arteriovenosa, Ultrassonografia Doppler

## Abstract

**Introduction:**

The increasing prevalence of chronic kidney disease has increased the demand for arteriovenous fistula (AVF) care. The objective of this study was to assess the relationship between some risk factors for AVF failure (advanced age, female sex, diabetes, obesity, central venous catheter, previous fistula, and hospitalization) and having a Doppler ultrasound performed preoperatively.

**Methods:**

A prospective study was performed with 228 dialysis patients from Imperatriz, Maranhão. Half of the sample was randomly selected to receive preoperative Doppler ultrasound and the other half did not, from the period of October 2016 to September 2018.

**Results:**

There were 53 total failures corresponding to 23.2% of our sample, which is almost double that of the patients in the clinical group. Considering the failures and risk factors associated with the overall sample, there was a statistically significant association between a central venous catheter on the same side of the AVF with P = 0.04 (Odds Ratio 1.24) and obesity with P = 0.05 (Odds Ratio 1.36), which was not repeated in the Doppler ultrasound group individually. There was no statistically significant difference between the Doppler group and clinical group with respect to the amount of days of previous AVF hospitalization and failure.

**Conclusions:**

We concluded that the reduction of failures with an introduction of the Doppler was statistically significant in the overall sample, but establishing a relationship between specific risk factors and failure was only possible with two of the risk factors in the study - obesity and central venous catheter on the same side of the AVF.

## RESUMO

**Introdução:**

A crescente prevalência de doença renal crônica aumentou a demanda por confecção de fístula arteriovenosa (FAV). O objetivo do presente estudo foi avaliar a relação entre alguns fatores de risco para falha da FAV (idade avançada, sexo feminino, diabetes, obesidade, cateter venoso central, fístula prévia e hospitalização) e a realização de ultrassonografia Doppler no pré-operatório.

**Métodos:**

Estudo prospectivo com 228 pacientes em diálise em Imperatriz, MA. Metade da amostra foi randomizada para receber ultrassonografia Doppler no pré-operatório. A outra metade dos pacientes não foi submetido a exame ultrassonográfico. O estudo incluiu pacientes atendidos no período de outubro de 2016 a setembro de 2018.

**Resultados:**

Houve 53 falhas (23,2%) em nossa amostra, quase o dobro do número dos pacientes no grupo clínico. Considerando as falhas e os fatores de risco associados à amostra geral, houve associação estatisticamente significativa entre catéter venoso central do mesmo lado da FAV (P = 0,04; Razão de Chances: 1,24) e obesidade (P = 0,05; Razão de Chances: 1,36), o que não foi reproduzido no grupo de ultrassonografia Doppler individualmente. Não houve diferença estatisticamente significativa entre o grupo Doppler e o grupo clínico em relação à quantidade de dias de internação e falha da FAV.

**Conclusão:**

A redução de falhas com a introdução do Doppler foi estatisticamente significativa na amostra geral, mas só foi possível estabelecer uma relação entre fatores de risco específicos e falha em dois dos fatores estudados, obesidade e catéter venoso central no mesmo lado da FAV.

## INTRODUCTION

The increased prevalence of chronic kidney disease (CKD) and consequent economic impacts on health services has resulted in a higher demand for arteriovenous fistula (AVF) care, which is considered to be the Achilles’ heel of hemodialysis.^1^ The Doppler ultrasound is a non-invasive method that enables safe structural and functional access to peripheral vessels, and is becoming the preferred image mode for AVF construction and follow-up.^1^^-^ 10

The Brazilian Society of Nephrology’s 2018 census data estimate 133,464 CKD dialysis patients in Brazil that year with the national prevalence being 640 pmp (patients per million population) and 276 pmp in Maranhão. The vast majority (92.3%) of dialysis patients are in hemodialysis.^11^

The autogenous arteriovenous fistula in the wrist has been the first choice for most surgeons, as it is the radio-cephalic fistula originally described by Brescia-Cimino in 1966 and still offers the least risk of complications as well as good durability.^12^^,^ 13 Grafts or catheters present a lower survival rate due to a higher incidence of thrombosis and occlusion by intimal hyperplasia, in addition to an increased susceptibility to infections, leading to greater patient morbimortality when compared with an autogenous arteriovenous fistula.^14^

According to the National Kidney Foundation - Kidney Disease Outcomes Quality Initiative (NKF-K/DOQI), an ideal access provides adequate flow rate, good durability, and has a low rate of complications (such as infection, stenosis, thrombosis, aneurysm, and limb ischemia). Among existing accesses, arteriovenous fistulas are the closest to ideal.^15^ Certain studies have shown an initial maturation rate to be unsatisfactory, with 30% to 60% failure in maturation.^16^^,^ 17

The Doppler ultrasound helps vascular surgeons plan the most suitable fistula configuration to optimize the rate of blood flow in the vascular access for hemodialysis, potentially reducing the incidence of arteriovenous fistulas dysfunction.^6^^,^ 18

The NKF-DOQI and the Society for Vascular Surgery’s Guidelines recommend image vessel mapping for all patients undergoing arteriovenous fistula for hemodialysis surgery.^15^^,^ 19

The use and interpretation of venous mapping for a preoperative Doppler ultrasound of AVFs vary considerably depending on the country, with high usage shown in the United States, regardless of patient characteristics. Canadian and European surgeons selectively use vessel mapping in patients with a BMI > 30, prior surgical access, history of central venous catheter use, and PICC - Peripherally Inserted Central Catheter.^20^

Our prospective study sought to analyze the relationship between risk factors for arteriovenous fistula failure (advanced age, female sex, diabetes, obesity, central venous catheter on the same side of the AVF, prior fistula, and performing the fistula soon after hospitalization) and having a Doppler ultrasound performed preoperatively.

## METHODS

The study was approved by the ABC-SP School of Medicine’s Ethics Committee (FMABC-SP) and is in accordance with the Declaration of Helsinki; all participants signed the informed consent form. This was a prospective study with dialysis patients from the hemodialysis clinics of Imperatriz-Maranhão (Clinic for Renal Disease and Imperatriz Nephrology Clinic) who provide services for the SUS (Unified Health System). The sample size was 228 patients. Half were randomly selected by a simple 1:1 draw to perform preoperative AVF Doppler ultrasound and the other half did not use this preoperative method and were only evaluated by physical examination. We evaluated the possible reduction of AVF failures associated with the following risk factors - advanced age, female sex, diabetes, obesity, central venous catheter on the same side as the AVF, previous fistula, and hospitalization close to AVF execution - and the use of Doppler. The study occurred from October 2016 to September 2018 with each patient being followed for 6 months (in the immediate postoperative period, at 1 week, 3 months, and 6 months).

The inclusion criteria were adult patients (age > 18 years) with a stable clinical condition, patent palmar arch (Allen test), and study authorization, luminal vessel diameter as described below, absence of stenosis or thrombosis in the central venous system, and absence of stenosis or arterial occlusion evaluated by Doppler ultrasound (ultrasound group). The exclusion criterion was patients who had the Doppler performed privately.

The ultrasound machine used was the HD11 XE Performance Plus^®^ (Philips) with transducer from 3 to 12 MHz, and the examination was performed by a single vascular sonographer. All patients were examined in a seated position, with their arms resting on the examination table. The scanning of superficial veins was performed with the use of a tourniquet. The compressibility of the cephalic and basilic veins across their path to B-mode, as well as the diameters of these veins were measured with a transverse section at the wrist, proximal 1/3 of the forearm, and distal and proximal 1/3 of the arm. The continuity of the deep venous system to the axillary and subclavian veins was evaluated. We investigated the diameter and flow of brachial, ulnar, and radial arteries, as well as subclavian and axillary arteries to evaluate possible stenosis. Patency of the palmar arch was evaluated via Allen test. The evaluation of the dominant arm was only performed when the non-dominant presented an unsatisfactory assessment.

In this study, the vessels met the minimum criteria of venous luminal diameter ≥ 2.5 mm for native fistulas (using tourniquet), axillary vein diameter ≥ 4.0 mm for arteriovenous grafts, and arterial luminal diameter ≥ 2.0 mm to be used in AVF implementation.^21^

Physical examination of the clinical group was performed by a vascular surgeon who constructed the AVFs. The veins were assessed with a tourniquet for diameter and compressibility, and edema or collateral circulation were evaluated in the arm with sign of central venous stenosis. The arterial segment was evaluated for pulsatility and Allen’s test.

The professional team consisted of 3 experienced vascular surgeons who provided their services to the SUS. Each surgeon performed the same amount of surgeries in both groups.

The AVF locations were mostly distal than proximal and in the non-dominant arm whenever possible. A prosthetic graft was used only when there was no native vein for AVF completion. When the AVF was performed, the surgeon evaluated the presence or absence of a thrill; however, nephrologists and skilled nurses clinically evaluated the AVF maturation. The AVFs and possible alterations were followed by 6 months. There was no endovascular procedure for fistula rescue performed in this study.

### DATA ANALYSIS

The collected data was stored in a Microsoft Excel 2016 spreadsheet. After checking for errors and inconsistencies, descriptive examinations were performed by means of absolute and relative frequencies and measures of central tendency and variability. 

The chi-square test, or equivalent, was used to assess associations between qualitative variables, and in the case of significant 2x2 associations, odds ratios and confidence intervals were calculated by means of logistic regression. For the analysis of quantitative variables, a Student’s t-test or similar non-parametric method was used. All examinations were performed at 5% significance level using the IBM SPSS^®^ program (IBM SPSS Statistics, Version 24.0, 2016).

## RESULTS

The main etiology of CKD was hypertension in both groups accompanied by diabetes, together corresponding to 90% of the causes of renal disease. Dominating factors were male sex, 60% of the cases, and age below 65 years, 78%, as shown in Table 1.

**Table 1:** Basic characteristics (n=228)

Characteristics		Percentage
Gender	Male	60%
	Female	40%
Age	< 65 years	78%
	≥ 65 years	22%
Etiology	Hypertension	50%
	Diabetes	40%
	Lupus	2.2%
	Multiple myeloma	0.9%
	Polycystic kidney disease	1.7%
	Unknown	2.6%
	Chronic glomerulonephritis	0.9%
	Other	1.7%
Diabetes	Yes	50.9%
	No	49.1%
Obesity	Yes	6.1%
	No	93.9%
Central venous catheter	Yes	15.8%
	No	84.2%
Prior AVF	Yes	36%
	No	64%

The data are presented as absolute frequency.

There were a total of 53 failures, corresponding to 23.2% of the total. Most (76%) occurred without effective use of the fistula (during negative exploration, immediate failure, and early thrombosis prior to maturity). In addition, 34% occurred in the ultrasound group, in contrast with 66% in the clinical group, as shown in Table 2.

**Table 2:** AVF failures (n=228)

Failures	Percentage	*P-*value
Clinical Group	66%	
Doppler Group	34%	
Early (Before maturation)	75.5%	0.02
Late (After maturation)	24.5%	

*P*-value of the Chi-squared test.

Considering the failures and risk factors associated with the overall sample (with and without Doppler), there was only significant association between having central venous catheter on the same side of the arteriovenous fistula with *P =* 0.04 (odds ratio of 1.24) and obesity with *P* = 0.05 (odds ratio of 1.36), as shown in Table 3. However, the Doppler group did not show significant association with any risk factor for failure, as set forth in Table 4.

**Table 3:** Correlation between failure and risk factors (n=228).

		Failure			Odds Ratio [95%CI]
		No	Yes	*P-*value*	
Gender	*Female*	68 (73.9%)	24 (26.1%)	0.40	-
	*Male*	107 (78.7%)	29 (21.3%)		-
Age	*< 65 years*	136 (76.4%)	42 (23.6%)	0.81	-
	*≥ 65 years*	39 (78.0%)	11 (22.0%)		-
Diabetes	*No*	86 (76.8%)	26 (23.2%)	0.99	-
	*Yes*	89 (76.7%)	27 (23.3%)		-
Obesity	*No*	167 (78.0%)	47 (22.0%)	0.05	Ref
	*Yes*	8 (57.1%)	6 (42.9%)		1.36 [1.01 – 2.16]
Central catheter	*No*	152 (79.2%)	40 (20.8%)	0.04	Ref
	*Yes*	23 (63.9%)	13 (36.1%)		1.24 [1.02 – 1.60]
Previous	*No*	110 (75.3%)	36 (24.7%)	0.50	-
AVF	*Yes*	65 (79.3%)	17 (20.7%)		-

* *P*-value of the Chi-square test.

**Table 4:** Ultrasound group: correlation between failure and risk factors (n=114).

		Failure		*P*-value
		No	Yes	
Gender	*Female*	42 (85.7%)	7 (14.3%)	0.70*
	*Male*	54 (83.1%)	11 (16.9%)	
Age	*< 65 years*	74 (82.2%)	16 (17.8%)	0.36**
	*≥ 65 years*	22 (91.7%)	2 (8.3%)	
Diabetes	*No*	45 (81.8%)	10 (18.2%)	0.50*
	*Yes*	51 (86.4%)	8 (13.6%)	
Obesity	*No*	91 (85.0%)	16 (15.0%)	0.31**
	*Yes*	5 (71.4%)	2 (28.6%)	
Central catheter	*No*	82 (83.7%)	16 (16.3%)	0.70**
	*Yes*	14 (87.5%)	2 (12.5%)	
Previous AVF	*No*	63 (79.7%)	16 (20.3%)	0.06**
	*Yes*	33 (94.3%)	2 (5.7%)	

* Chi-square test.

** Fisher Test.

Table 5 shows the Doppler data with the venous diameters and depths, as well as arterial diameters, considering that the venous inclusion criteria were a diameter of ≥ 2.5 mm, arterial of ≥ 2.0 mm, and axillary of ≥ 4.0 mm. Independent of the depth of the basilic vein in the upper arm, it was transposed anteriorly in all patients of the two groups.

**Table 5:** Means and standard deviation in the preoperative ultrasound mapping group (n=114).

Doppler Ultrasound		MEAN	SD
Cephalic vein (n=78)	*Diameter (cm)*	0.37	0.08
	*Depth (cm)*	0.19	0.09
Basilic vein (n=23)	*Diameter (cm)*	0.40	0.11
	*Depth (cm)*	0.64	0.31
Axillary vein (n=13)	*Diameter (cm)*	0.69	0.14
Radial artery (n=29)	Diameter (cm)	0.23	0.03
	*PSV (cm/sec)*	52.93	17.50
Brachial artery (n=85)	*Diameter (cm)*	0.39	0.09
	*PSV (cm/sec)*	68.00	21.27

SD: standard deviation.

No significant correlation was observed between number of hospitalization days and failure rate in both groups and there was no difference in hospitalization time between the Doppler and clinical group, as shown in Table 6.

**Table 6:** Number of days hospitalized as per group and failure rate of patients undergoing AVF (n=228).

Days hospitalized (Mean ± Standard Deviation)			*p* *
Group	Clinical (n=114)	8.91±13.40	0.052
	Clinical (n=114)	10.98±14.34	
Failures	Yes (n=53)	9.34±13.58	0.29
	No (n=175)	11.94±14.80	

* Nonparametric Wilcoxon-Mann-Whitney *U* test.

## DISCUSSION

In our trial, hypertension was the main cause of CKD followed by diabetes, and showed predominance in males, which is in agreement with several other studies.^2^^,^ 11 ^,^ 22 ^-^ 27 Compared to previous studies, we found a slightly lower failure rate.^22^^,^ 28 ^-^ 30

We found no statistically significant association between fistula failures and risk factors such as advanced age, female gender, and diabetes, as reported in some studies.^24^^,^ 31 ^-^ 36 However, despite our initial sample of 228 patients, failure occurred in only 53 cases, reducing our sample for association analysis, which might explain the lack of association as suggested by the studies cited above.

In the overall sample, patients with obesity were 1.36 times more likely to experience failure than the non-obese; those with a central catheter were 1.24 times more likely to experience failure than those who without. In the Doppler ultrasound group, there was no significant association with a central catheter or obesity, which suggests that the Doppler provides for a reduction of failures in these patients.

There was no difference between the number of days of hospitalization between the groups who experienced failures or not (p=0.29), and there was no difference between the number of days of hospitalization among the clinical and ultrasound groups (p=0.052). This shows that both the physical examination and the Doppler ultrasound were able to identify the veins damaged by possible punctures before the AVF. We did not find similar data in the literature for comparison.

## CONCLUSION

We conclude that the reduction of failure rate with the use of the Doppler was statistically significant in the overall sample; nonetheless, establishing a relationship between specific risk factors and failures was only possible with two risk factors - obesity and a central venous catheter on the same side of the AVF. This shows the benefit of preoperative use of Doppler in AVFs, mainly to reduce failures related to these two risk factors. Further studies with larger sample sizes are recommended.
